# Calcium carbonate-enriched pumpkin affects calcium status in ovariectomized rats

**DOI:** 10.1007/s13197-023-05686-3

**Published:** 2023-02-10

**Authors:** Natalia Wawrzyniak, Anna Gramza-Michałowska, Paweł Kurzawa, Paweł Kołodziejski, Joanna Suliburska

**Affiliations:** 1 Department of Human Nutrition and Dietetics, Faculty of Food Science and Nutrition, Poznań University of Life Sciences, 31 Wojska Polskiego St., 60-624 Poznan, Poland; 2 Department of Gastronomy Science and Functional Foods, Faculty of Food Science and Nutrition, Poznań University of Life Sciences, Wojska Polskiego 31, 60-624 Poznan, Poland; 3 Department of Clinical Pathology, Poznań University of Medical Sciences, Przybyszewskiego 49, 60-355 Poznan, Poland; 4 Department of Oncological Pathology, Pozna University of Medical Sciences, Szamarzewskiego 84, 60-596 Poznan, Poland; 5 Department of Animal Physiology, Biochemistry and Biostructure, Faculty of Veterinary Medicine and Animal Science, Poznań University of Life Sciences, Wojska Polskiego 28, 60-637 Poznan, Poland

**Keywords:** Calcium, Enriched pumpkin, Osteoporosis, Ovariectomy, Rat

## Abstract

Calcium carbonate (CaCO_3_)-enriched pumpkin may serve as a good source of calcium for patients diagnosed with osteoporosis. In this study, we aimed to determine the effect of CaCO_3_-enriched pumpkin on Ca status in ovariectomized rats. The study included 40 female Wistar rats divided into five groups (n = 8). One group was fed with a standard diet (control group), while the other four groups were ovariectomized and received a standard diet (control ovariectomized group), or a diet containing CaCO_3_-enriched pumpkin, alendronate, or both. The nutritional intervention lasted 12 weeks, and then the rats were euthanized. Tissue and blood samples were collected and assessed for the levels of total Ca, estradiol, parathyroid hormone, and procollagen type I N propeptide. In addition, a histological analysis was performed on femurs. The results of the study suggest that CaCO_3_-enriched pumpkin can increase Ca content in femurs and improve bone recovery in ovariectomized rats. Furthermore, enriched pumpkin contributes to Ca accumulation in the kidneys, and this effect is more pronounced in combination with alendronate.

## Introduction

Postmenopausal osteoporosis is a condition characterized by a reduction in bone mineral mass due to a decline in estrogen levels as a result of endocrine disruption of the ovaries (Black and Rosen 2016). It is mainly diagnosed using dual X-ray absorptiometry (Yong and Logan 2021), but serum bone markers are also considered one of the prognostic indicators to determine disease development or treatment effects (Kanis et al. 2020). Bone turnover markers can be divided into two groups: formation markers and resorption markers. The former includes PINP and OC, which are by-products of bone mineralization. PINP found in the serum is released during collagen formation, while PTH stimulates osteoblasts to release OC (Marcu et al. 2011; Greenblatt et al. 2017). Histopathological analysis of bone aids in understanding the bone structure and cellular changes occurring in the bone tissue, thereby confirming the presence or absence of osteoporotic changes. A fewer number of osteoblasts and osteocytes (forming cells) and an increased number of osteoclasts (resorbing cells) might indicate the presence of osteoporosis. Moreover, a higher ratio of fat bone marrow to bone marrow cellularity is a negative prognostic indicator of osteoporosis development (Marcu et al. 2011). The percentage of de novo-built bones is indicated by the percentage of woven bones. First, a woven bone (immature bone undergoing reconstruction) is formed from mesenchymal osteoblasts; the woven bone is then remodeled into a lamellar bone (mature bone that does not undergo transformation) from surface osteoblasts, a process common in the general population. However, the proportion of woven bone to lamellar bone varies among individuals (Shapiro and Wu 2019). The number of woven bones is generally high during recovery from injury or during growth in children, whereas in adults bone formation and resorption processes occur continuously and bones undergo standard transformations (Downey and Siegel 2006).

The diagnosis of osteoporosis should be followed by appropriate treatment to increase bone density and decrease bone turnover. Pharmacological treatment intended for osteoporosis involves the use of drugs that reduce bone resorption and/or accelerate bone formation, such as bisphosphonates (alendronate and risedronate), denosumab, and teriparatide, or hormone replacement (Gallagher and Tella 2014). However, these drugs can cause side effects when used for a long term; for example, the use of bisphosphonates for over two years can result in atypical bone fractures (Black and Rosen 2016), jaw necrosis (Shibahara 2019), or digestive disorders (Fadda et al. 2015). Therefore, the public health system is currently focusing on developing new approaches for the treatment and prevention of osteoporosis.

A diet containing adequate amounts of Ca with high bioavailability is essential for maintaining bone health, as Ca constitutes a large portion of bone mass (Weaver 2015). In addition to eliminating substances that can reduce Ca absorption (e.g. phytic or oxalic acid), components that increase Ca bioavailability (e.g. vitamin D and inulin) should be included adequately in the diet (Wawrzyniak and Suliburska 2021), in order to improve bone health. Endogenous factors regulating the metabolism of Ca are equally important. The concentration of Ca in the blood is regulated mainly by active vitamin D (1,25-dihydroxycholecalciferol), calcitonin, and PTH. PTH plays a major role in Ca regulation in the blood and bone turnover, which stimulates the release of Ca in bones and its reabsorption in kidneys. In addition, PTH stimulates the synthesis of vitamin D, which increases intestinal Ca uptake, and inhibits collagen synthesis by osteoblasts. Collagen is the organic matrix for minerals (including Ca) deposited in bone. On the contrary, calcitonin inhibits bone resorption, the activation of vitamin D, and Ca renal reabsorption. Therefore, the status of Ca in the body is influenced by the supply, bioavailability, and factors regulating its metabolism (Marcu et al. 2011; Greenblatt et al. 2017).

Epidemiological studies have shown that Ca deficiency is common worldwide, and there is a need to identify dietary sources with high bioavailable Ca (Balk et al. 2017). It has been found that Ca-enriched food products based on pumpkin can help overcome this challenge (Weaver and Liebman 2002). Pumpkin comprises compounds with high biological activity, such as carotenoids, including α-carotene, β-carotene, zeaxanthin, or lutein, which have a beneficial effect on the bone mineral status, reduce susceptibility to fractures, and prevent the progression of osteoporosis (Kulczynski and Gramza-Michałowska 2019). Moreover, pumpkin is easy to use in an osmotic dehydration process, which allows the enrichment of its tissues with Ca salts (Kulczyński et al. 2020). Enriched pumpkin contains inulin which increases Ca bioavailability(Bakirhan and Karabudak 2021), other ingredients that can improve bone health, including lutein (Takeda et al. 2017; Tominari et al. 2017) and β-cryptoxanthin, and a pigment that inhibits bone resorption and reduces oxidative stress (Yamaguchi 2012; Ozaki et al. 2015). Pumpkin has also been used in previous studies due to its low caloric content (average 26 kcal/100 g). Moreover, pumpkin exhibits cardioprotective and hypoglycemic properties, and is therefore recommended for diabetics and patients with arterial hypertension and obesity (Kulczynski and Gramza-Michałowska 2019).

According to the available data, the ingredients of Ca-enriched pumpkin can increase bone mineral density and thus reduce the risk of osteoporosis. Therefore, this study aimed to determine the effect of Ca-enriched pumpkin on Ca metabolism and status in ovariectomized rats.

## Methods

### Materials and reagents

Pumpkins (*Cucurbita maxima*, yellow melon) were obtained from a domestic organic farm after seeking permission from the land owner. Experimental research and field studies, including the collection of plant materials, were conducted in accordance with relevant institutional, national, and international guidelines and legislation. Inulin and CaCO_3_ were purchased from Agnex (Białystok, Poland). Sucrose, rapeseed oil, dextrin, corn starch, and casein were obtained from Hortimex (Konin, Poland). Minerals and vitamins were procured from Sigma-Aldrich (Darmstadt, Germany). ELISA kits were purchased from SunRed (Shanghai, China).

### Osmotic dehydration

Pumpkins were enriched with CaCO_3_ by osmotic dehydration with inulin, an osmotically active substance. Then, they were cleaned and washed, and the inner part attached to the seeds was removed. Subsequently, the skin was peeled, and the pumpkins were cut into cubes (1 cm) and frozen for 24 h at − 18 °C. After freezing, a solution composed of inulin (125 g) and distilled water (125 ml) in a 1:1 ratio was prepared in small jars. CaCO_3_ was added to the prepared solution such that its content was 5% of the solution. Next, 50 g of frozen pumpkin cubes (5:1) was added to 250 g of the hypertonic solution in the jars. The jars containing the pumpkin cubes were closed and shaken for 2 h at 50 °C in a water bath. Upon completion of osmotic dehydration, the solution formed above the pumpkin cubes was removed and the cubes were filtered. This process was repeated three times. Then, the pumpkin cubes were frozen at − 18 to − 28 °C for the next 24 h, and freeze-dried to 3.5–5% water content. A 100 g of the obtained lyophilizate contained 2390.8 ± 63.3 mg of Ca (Kulczyński et al. 2020) compared to nonenriched freeze-dried pumpkin, in which the Ca content was only 264.89 ± 0.59 mg/100 g (Kulczynski and Gramza-Michałowska 2019).

### Animals

Forty female Wistar rats aged 12 weeks were purchased from the University of Adam Mickiewicz in Poznań, Greater Poland Center for Advanced Technologies, Poland. The animals were allowed to acclimatize for 1 week and then housed individually in cages under a 12-h dark–light cycle. All animal experiments were carried out in accordance with the EU Directive 2010/63/EU for animal experiments. Approval for the study was obtained from the Local Ethics Committee in Poznań (protocol number: 34/2019). The reporting in the manuscript follows the recommendations in the ARRIVE guidelines.

### Experimental protocols

**Fig. 1:**
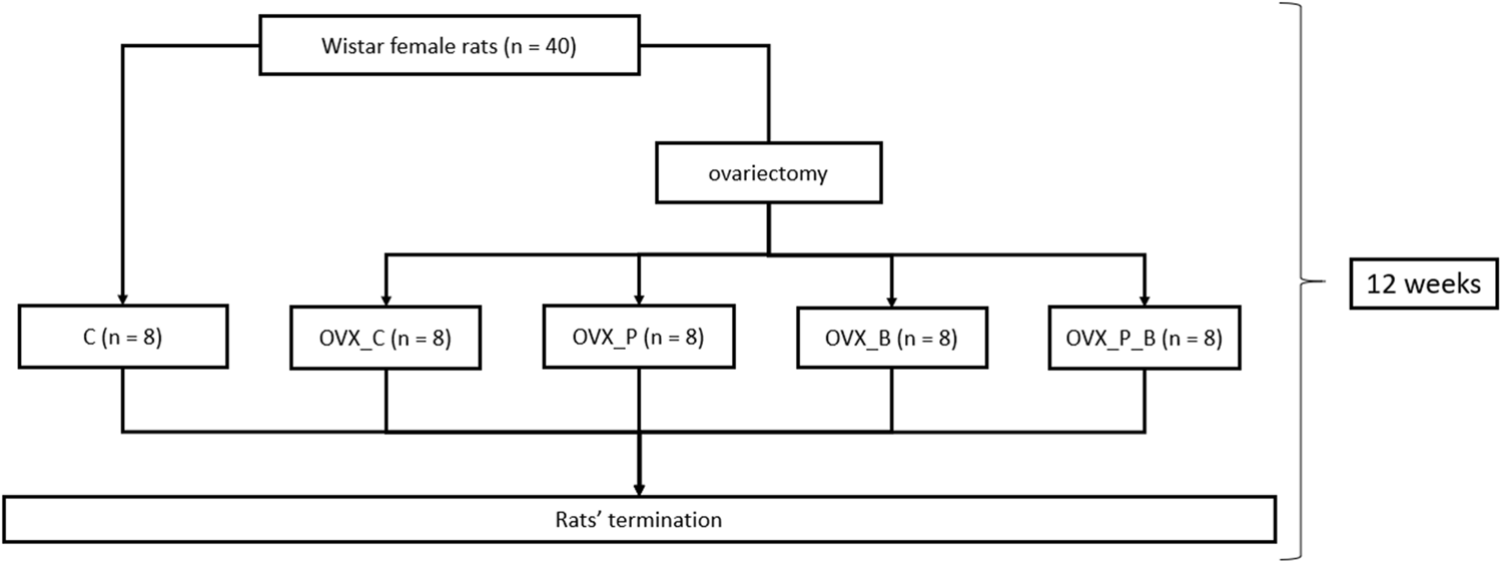
Scheme of the study. C, control group receiving standard diet; OVX_C, ovariectomized group receiving standard diet; OVX_P, ovariectomized group receiving diet with pumpkin enriched with CaCO3; OVX_B, ovariectomized group receiving diet with alendronate; OVX_P_B, ovariectomized group receiving diet with pumpkin enriched with CaCO_3_ and alendronate

Throughout the experiment, the rats were fed with the standard AIN-93 M diet (Reeves and Suppl 1997). The animals were divided into five groups, with eight in each. At the beginning of the experiment, the body weight of the rats was measured and found to be similar. Four groups (32 rats) were ovariectomized. After a one-week recovery period, the rats were subjected to a 12-week nutritional intervention. Unmodified standard AIN-93 M diet was given to the control group (C) and to one of the ovariectomized groups (OVX_C), while the other three groups received a diet containing CaCO_3_-enriched pumpkin (OVX_P group), alendronate (OVX_B), or alendronate and CaCO_3_-enriched pumpkin (OVX_P_B group). The standard diet contained CaCO_3_ as a source of Ca. Figure 1 presents a schematic of the experiment.

The amount of enriched pumpkin added to the modified diet was such that the Ca content of the modified diet was the same as that of the standard diet. For the OVX_B and OVX_P_B groups, the amount of alendronate was adjusted weekly, ensuring that they received 3 mg/kg body weight. All animals were allowed *ad libitum* access to deionized water and feed. The intake by animals was recorded daily, and the body weight was measured weekly. At the end of the experiment, a body weight was measured, and then the rats were decapitated. Blood samples were collected and stored at − 80 °C. Serum was obtained by centrifuging the blood samples at 1200 × g for 10 min at 4 °C. The femurs, liver, kidneys, femoral muscles, spleen, and pancreas were isolated for analyses. The obtained tissues were washed with saline, weighed, and stored at − 80 °C. Hair was collected from the interscapular area.

### Diet analysis

The lipid content in the samples was determined using the Soxhlet method (PN-EN ISO 3947:2001; Soxtec System, Foss Tecator), while the protein content was determined using the Kjeldahl method (AOAC, 1995; Foss Tecator). The sample was completely burned in a muffle furnace to determine the ash content (AOAC, 2000). The carbohydrate content was calculated from the content of fat, protein, water, and ash. The total fiber fraction was measured using the enzymatic-gravimetric method (Dziedzic et al. 2012).

### Ca analysis in diets

To determine Ca content in diets, 1 g of each sample of diet was burned in a muffle furnace at 450 °C until mineralization. The samples were then dissolved in 1 mol/l nitric acid (Merck, Kenilworth, NJ, USA). Using flame atomic absorption spectrometry, the mineral content was determined after diluting the samples with appropriate amounts of LaCl_3_ (0.5%) and deionized water (AAS-3, Carl Zeiss, Jena, Germany). The method was validated with an accuracy of 92% using brown bread (BCR191, Sigma-Aldrich, St. Louis, MO, USA), a certified reference material. All diet samples were analyzed in triplicate.

### Ca analysis in tissues

To determine Ca content in tissues, the samples were mineralized in a microwave digestion system (Speedwave Xpert, Berghof, Eningen, Germany) with pure nitric acid (Merck, Kenilworth, NJ, USA). After digestion, the samples were mixed with deionized water and then diluted with LaCl_3_ (0.5%). The concentration of minerals was determined by flame atomic absorption spectrometry (AAS-3, Carl Zeiss, Jena, Germany) at a wavelength of λ = 422.7 nm. The method was validated with an accuracy of 91% using bovine liver (1577 C, Sigma-Aldrich, St. Louis, MO, USA), a certified reference material.

### Histological analysis

The resected femoral bones were fixed with 10% buffered formalin for 24 h. Then, the specimens were decalcified in Osteodec bone marrow biopsy decalcifying solution for another 3 h. Subsequently, each specimen was routinely processed and embedded separately in paraffin blocks. Two-micrometer-thick sections were cut from the blocks (three sections for each tissue sample) and stained with hematoxylin and eosin. Each slide contained two femoral bone sections with the bone marrow content. The bone marrow of each bone was analyzed under a light microscope (Leica, Allendale, NJ, USA), and the content of adipose tissue in the bone marrow was assessed separately by two scientists under a high-power field (HPF; 400× magnification). The percentile amount of fat bone marrow in the bone marrow was estimated under a light microscope in five different HPF areas (400× magnification, area of 0.25 mm^2^), and the mean value was calculated. The number of osteoblasts, osteocytes, and osteoclasts was counted in each HPF area (400× magnification, area of 0.25 mm^2^). The percentile amount of woven bone was also estimated under a light microscope in five different HPF areas (400× magnification, area of 0.25mm^2^), and the mean percentile amount in the entire bone was calculated.

### Biochemical parameters

The serum concentrations of PTH, PINP, OC, and ES were determined by ELISA using commercial ELISA kits (SunRed, Shanghai, China) which are used to estimate the mentioned parameters in samples of rat serum, blood, and plasma, and in other related tissue liquids. The precision of the technique used was validated, and the intra-assay and inter-assay precision (CV (%) = SD/mean × 100) for the estimation of ES, PTH, PINP, and OC was found to be < 9% and < 11%, respectively. The sensitivity of the method for each determined parameter was as follows: 3.112 ng/l for ES, 0.227 ng/dl for PTH, 0.325 ng/ml for PINP, and 0.523 ng/ml for OC. The analysis was carried out using an infinite F50 spectrometer (Tecan Group Ltd., Männedorf, Switzerland). The ELISA kits used were based on the principle of the dual antibody sandwich technique for the detection of parameters in rats’ materials.

### Statistical analysis

Statistical analysis was performed using the Statistica program (StatSoft, Tulsa, OK, USA). The normality of the distribution of the variables was determined using the Shapiro–Wilk test. Statistical differences between the analyzed groups were determined using a one-way analysis of variance with Tukey’s post hoc test. *P-*value < 0.05 was considered statistically significant. The results are presented as mean values ± standard deviation.

## Results

Table 1 shows the composition of the standard diet provided to groups C, OVX_C, and OVX_B and that of the diet with enriched pumpkin provided to groups OVX_P and OVX_P_B. The content of macronutrients and Ca was comparable in both diets.

**Table 1:** Composition of diets (mean *±* standard deviation)

Components	Diets		
	Standard (C/OVX_C/OVX_B groups)	Enriched pumpkin (OVX_P/OVX_P_B groups)	
Caloric value (kcal/100 g)	326.37 ± 4.48	311.59 ± 3.29	
Carbohydrates (g/100 g)	47.92 ± 0.60	41.6 ± 2.06	
Fiber (g/100 g)	23.04 ± 0.60	23.92 ± 0.69	
Fat (g/100 g)	3.76 ± 0.41	4.17 ± 0.38	
Protein (g/100 g)	13.70 ± 0.21	14.95 ± 1.6	
Ca (mg/g)	5.63 ± 0.37	5.57 ± 0.38	

*C* control group, *OVX_C* ovariectomized group, *OVX_B* ovariectomized group receiving alendronate; *OVX_P* ovariectomized group receiving pumpkin enriched with CaCO_3_, *OVX_P_B* ovariectomized group receiving pumpkin enriched with CaCO_3_ and alendronate; alendronate concentration:3 mg/kg body weight

Ovariectomy causes changes in body composition and in estrogen levels. In this study, ovariectomized rats showed a significant increase (*P* < 0.05) in body mass (Table 2). However, modified diets did not significantly (*P* > 0.05) affect the weight of rats in the ovariectomized groups (Table 2). As expected, ovariectomy also caused a significant decrease (*P* < 0.05) in serum ES concentration in rats (Table 3). An analysis of the parameters of Ca metabolism was also performed in this study, and the results are presented in Table 3. It was observed that ovariectomy had no significant effect (*P* > 0.05) on the concentration of PINP, while the combination of alendronate and enriched pumpkin caused a significant increase (*P* < 0.05) in the level of this bone formation marker in comparison to the control group. Similarly, ovariectomy did not significantly (*P* > 0.05) influence affect the PTH levels in the serum of rats. However, the addition of enriched pumpkin and alendronate alone in the diet caused an increase in PTH levels in rats in comparison to the OVX_C group, but this effect was not observed in rats that received the diet containing both these substances (OVX_P_B group).

**Table 2:** Daily intake and body mass in rats (mean *±* standard deviation)

Parameter	Group					
	C	OVX_C	OVX_P	OVX_B	OVX_P_B	
Daily intake of diet (g)	24.98 ± 0.54	25.17 ± 1.49	23.74 ± 0.97	24.76 ± 0.28	24.19 ± 1.3	
Daily intake of Ca (mg)	140.53 ± 3.05	141.61 ± 8.4	133.94 ± 3.05	142.39 ± 1.61	136.19 ± 7.31	
Body mass (g)	338.59 ± 29.71^a^	442.71 ± 29.63^b^	430.31 ± 14.94^b^	439.01 ± 29.07^b^	424.05 ± 30.19^b^	

*C* control group, *OVX_C* ovariectomized group, *OVX_P* ovariectomized group receiving pumpkin enriched with CaCO_3_; *OVX_B* ovariectomized group receiving alendronate, *OVX_P_B* ovariectomized group receiving pumpkin enriched with CaCO_3_ and alendronate; alendronate concentration:3 mg/kg body weight

^a,b^Significant differences between groups (*p* < 0.05)

**Table 3:** Level of estradiol and parameters of Ca metabolism in serum of rats (mean *±* standard deviation)

Parameter	Group				
	C	OVX_C	OVX_P	OVX_B	OVX_P_B
ES (ng/l)	49.88 ± 5.06^b^	23.39 ± 5.55^a^	22.03 ± 5.27^a^	18.06 ± 2.65^a^	18.79 ± 3.59^a^
PINP (ng/ml)	3.55 ± 0.83^a^	4.6 ± 0.86^ab^	4.6 ± 0.5^ab^	4.58 ± 1.03^ab^	5.17 ± 1.03^b^
PTH (ng/dl)	3.44 ± 0.48^ab^	2.56 ± 0.48^a^	3.54 ± 0.71^b^	3.58 ± 0.74^b^	3.12 ± 0.42^ab^
OC (ng/ml)	18.57 ± 3.8	16.28 ± 1.17	17.56 ± 4.86	18.5 ± 6.05	16.39 ± 4.25

*C* control group, *OVX_C* ovariectomized group, *OVX_B* ovariectomized group receiving alendronate, *OVX_P* ovariectomized group receiving pumpkin enriched with CaCO_3_, *OVX_P_B* ovariectomized group receiving pumpkin enriched with CaCO_3_ and alendronate; alendronate concentration:3 mg/kg body weight

^a,b^ significant differences between groups (*p* < 0.05)

To estimate the effect of modified diets on the Ca status in rats, the content of this element was estimated in the collected tissue samples and serum (Table 4). It was found that ovariectomy caused a significant reduction (*P* < 0.05) in Ca content in the femur. In turn, the addition of enriched pumpkin to the diet increased the femoral Ca concentration, and a similar effect was observed in the group that received the diet with alendronate. The addition of alendronate and enriched pumpkin (OVX_P_B group) in combination also caused an increase in Ca concentration in the femur; however, this effect was less pronounced than that observed with the use of enriched pumpkin (OVX_P group) and alendronate (OVX_B group) alone. Ovariectomy caused a significant reduction (*P* < 0.05) in Ca content in the heart. The concentration of Ca in the spleen was significantly lower (*P* < 0.05) in the OVX_B and OVX_P_B groups compared to the OVX_C group. In the OVX_P group, a significant decrease (*P* < 0.05) in the Ca level was observed in the liver in comparison to the OVX_C group. Ovariectomy had no effect on Ca content in muscles, while the addition of alendronate alone and in combination with enriched pumpkin to the diet caused a significant reduction (*P* < 0.05) in the muscle Ca content. The use of modified diets led to a significant increase (*P* < 0.05) in the Ca content in the kidneys (almost twofold in the OVX_P group, threefold in the OVX_B group, and fivefold in the OVX_P-B group). The diet containing both alendronate and enriched pumpkin promoted more Ca accumulation in the kidneys than the diet containing either of these components.

**Table 4:** Ca content in serum and tissues (mean *±* standard deviation)

Parameter	Group					
	C	OVX_C	OVX_P	OVX_B	OVX_P_B	
Serum (µg/ml)	132.65 ± 12.81	121.3 ± 8.76	112.94 ± 7.03	115.9 ± 8.48	124.43 ± 18.43	
Femur (mg/g dm)	239.28 ± 18.01^b^	217.22 ± 8.16^a^	279.17 ± 12.83^c^	290.94 ± 15.55^c^	256.96 ± 10.22^b^	
Pancreas (µg/dm)	110.11 ± 12.19	114.2 ± 11.27	102.89 ± 18.82	97.79 ± 10.97	108.15 ± 11.98	
Hair (µg/g dm)	603.68 ± 170.1^b^	463.87 ± 55.87^ab^	369.4 ± 78.73^a^	413.12 ± 99.01^a^	451.46 ± 54.8^a^	
Spleen (µg/g dm)	527.47 ± 112.62^c^	460.31 ± 98.7^c^	425 ± 54.04^bc^	347.39 ± 27.01^ab^	289.1 ± 33.07^a^	
Liver (µg/g dm)	156.01 ± 9.01^bc^	140.74 ± 11.84^bc^	94.91 ± 12.37^a^	134.84 ± 21.87^b^	159.54 ± 15.44^c^	
Heart (µ/g dm)	118.55 ± 16.79^b^	83.9 ± 8.14^a^	80.98 ± 7.47^a^	81.5 ± 11.38^a^	72.41 ± 8.67^a^	
Brain (µg/g dm)	178.93 ± 27.45	227.33 ± 85.69	219.14 ± 81.91	196.76 ± 86.43	239.07 ± 85.09	
Muscle (µg/g dm)	47.4 ± 4.08^c^	52.81 ± 11.46^c^	42.9 ± 4.91^bc^	34.37 ± 5.4^ab^	25.25 ± 6.36^a^	
Kidney (µg/g dm)	91.19 ± 11.26^a^	79.36 ± 8.19^a^	148.66 ± 29.68^b^	257.4 ± 42.85^c^	461.07 ± 40.67^d^	

*C* control group, *OVX_C* ovariectomized group, *OVX_B* ovariectomized group receiving alendronate, *OVX_P* ovariectomized group receiving pumpkin enriched with CaCO_3_, *OVX_P_B* ovariectomized group receiving pumpkin enriched with CaCO_3_ and alendronate; alendronate concentration:3 mg/kg body weight; dm, dry mass

^a,b,c,d^Significant differences between groups (*p* < 0.05)

**Fig. 2:**
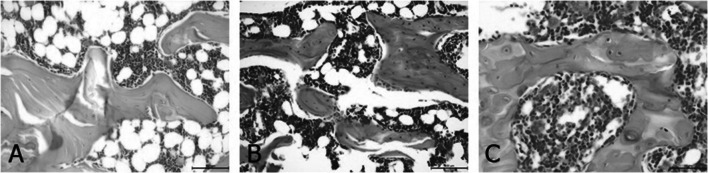
Differences between the number of osteoblasts: **A** few osteoblasts along the bones in the representative of the OVX_C group (H&E; 100×); **B** numerous clusters of osteoblasts arranged along the bones in the representative of the OVX_B group (H&E; 100×); **C** numerous clusters of osteoblasts arranged along the bones in the representative of the OVX_P_B group (H&E; 100×)

**Fig. 3:**
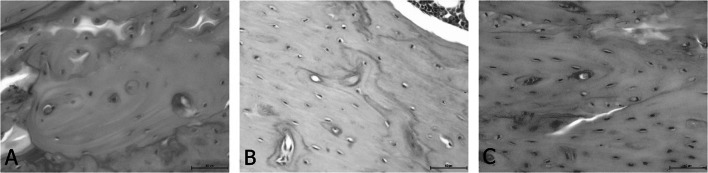
Differences between the number of osteocytes: **A** few osteocytes in the bone in the representative of the C group (H&E; 200×); **B** average number of bone osteocytes in the representative of the OVX_C group (H&E; 200×); **C** large number of bone osteocytes in the representative of the OVX_B group (H&E; 200×)

**Fig. 4:**
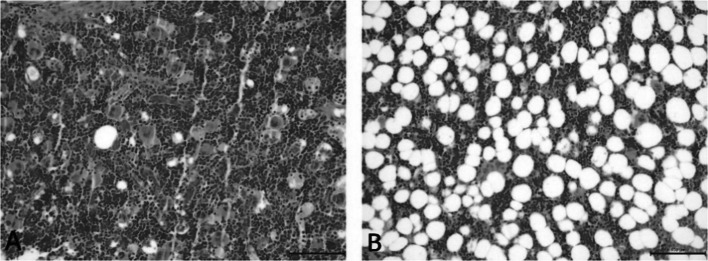
Differences between the amount of bone marrow femoral adipocytes: **A** low number of bone marrow femoral adipocytes in the representative of the C group (H&E; 400×); **B** several number of bone marrow femoral adipocytes in the representative of the OVX_C group (H&E; 400×)

**Fig. 5:**
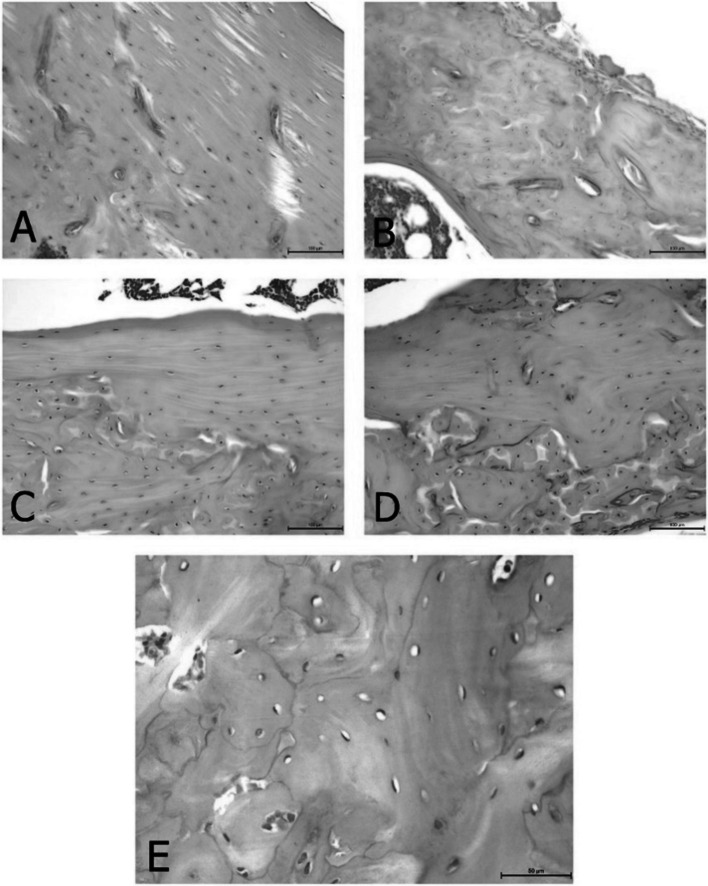
Differences between the content of woven bones: **A** low content of woven bone in the representative of the C group (H&E; 200×); **B** high content of woven bone in the representative of the OVX_C group (H&E; 200×); **C** average content of woven bone in the representative of the OVX_P group (H&E; 200×); **D** average content of woven bone in the representative of the OVX_B group (H&E; 200×); **E** high content of woven bone in the representative of the OVX_P_B group (H&E; 400×)

To assess bone structure and bone health related to Ca metabolism, the study also included a histological analysis of the femur in rats (Table 5). The changes observed in the bone structure are presented in Figs. 2, 3, 4 and 5. It was found that ovariectomy did not affect the numbers of osteoblasts and osteocytes, but the addition of alendronate with or without enriched pumpkin to the diet caused a significant increase (*P* < 0.05) in these parameters in rats in comparison to the OVX_C group. Moreover, ovariectomy reduced the number of osteoclasts and increased fat bone marrow, but modified diets did not reverse this effect. Ovariectomy also caused an increase in the percentage of woven bone, but alendronate and enriched pumpkin, even when used alone, reversed this effect.

**Table 5:** Parameters of the histological analysis of the femur (mean *±* standard deviation)

Parameter	Group				
	C	OVX_C	OVX_P	OVX_B	OVX_P_B
Number of osteoblasts	10 ± 3.78^a^	10.5 ± 5.13^a^	16 ± 7.01^ab^	20 ± 4.5^b^	18.63 ± 5.93^b^
Number of osteocytes	38.75 ± 8.35^a^	45 ± 8.86^ab^	40.25 ± 11.85^a^	57.5 ± 8.02^bc^	61.38 ± 11.99^c^
Number of osteoclasts	0.88 ± 0.99	0 ± 0	0 ± 0	0.25 ± 0.46	0 ± 0
Bone marrow fat (%)	8.13 ± 3.72^a^	43.75 ± 10.61^b^	46.25 ± 9.16^b^	36.25 ± 5.18^b^	38.75 ± 6.41^b^
Woven bone (%)	8.13 ± 2.59^a^	18.75 ± 8.35^c^	10 ± 4.63^ab^	11.25 ± 3.54^ab^	16.88 ± 3.72^bc^

*C* control group, *OVX_C* ovariectomized group, *OVX_B* ovariectomized group receiving alendronate, *OVX_P* ovariectomized group receiving pumpkin enriched with CaCO_3_, *OVX_P_B* ovariectomized group receiving pumpkin enriched with CaCO_3_ and alendronate, alendronate concentration:3 mg/kg body weight

^a,b,c^Significant differences between groups (*p* < 0.05)

The study also analyzed the relationships between the examined parameters, and the results of the correlation analysis are presented in Table 6. A significant negative correlation (*P* < 0.05) was found between body mass and serum ES level (*r* = − 0.67) as well as between body mass and serum Ca concentration (*r* = − 0.51). Similarly, a negative correlation in Ca level was found between the kidney and the spleen (*r* = − 0.73), between the kidney and muscle (*r* = − 0.93), and between the femur and the pancreas (*r* = − 0.56). A negative correlation was also found between the Ca content in muscles and the P1NP level in serum (*r* = − 0.62). A positive correlation was observed between the PTH level in serum and the Ca level in the femur (*r* = 0.64).

**Table 6:** Significant (*P* < 0.05) Pearson correlation coefficient (*r*)

Parameters	*r*
Body mass (g) and ES (ng/l)	–0.67
Body mass (g) and Ca in serum (µg/ml)	–0.51
Ca in kidney (µg/g dm) and Ca in spleen (µg/g dm)	–0.73
Ca in kidney (µg/g dm) and Ca in muscle (µg/g dm)	–0.93
Ca in femur (mg/g dm) and Ca in pancreas (µg/g dm)	–0.56
PINP (ng/ml) and Ca in muscle (µg/g dm)	–0.62
PTH (ng/dl) and Ca in femur (mg/g dm)	0.64

dm, dry mass

## Discussion

The results of the study showed that CaCO_3_-enriched pumpkin increased bone Ca content to the same extent as alendronate. This is a valuable finding as it may indicate that Ca combination with pumpkin can prevent bone resorption and contribute to an increase in bone formation. Because the Ca content was comparable in the tested diets, some ingredients in pumpkin could have improved Ca bioavailability from enriched pumpkin, which contains large amounts of inulin, and Ca metabolism, which might affect bone structure. It has been shown that inulin can improve Ca bioavailability in the intestine and can stimulate the transport of active Ca ions to cells, probably by increasing the level of calbindin (a transport protein) (Nzeusseu et al. 2006; Bakirhan and Karabudak 2021). Furthermore, from this study, it seems that Ca ions are shifted between tissues and that these ions may accumulate in bones at the expense of other tissues (Li et al. 2019; He et al. 2020). This mechanism was partly confirmed by the inverse correlation observed between Ca content in the pancreas and femur. Moreover, pumpkin contains other ingredients such as carotenoids, zeaxanthin, lutein, which could prevent bone resorption in rats after ovariectomy (Yamaguchi 2012; Ozaki et al. 2015; Takeda et al. 2017; Tominari et al. 2017). However, the changes observed in Ca content in the femur are difficult to associate with PTH levels. Although the levels of PTH were not changed by ovariectomy, the addition of alendronate and enriched pumpkin to the diet contributed to an increase in this hormone. The results obtained for PTH concentration in ovariectomized groups were unexpected. It is challenging to directly explain the low PTH concentration observed in the OVX_C group and the relatively high concentrations observed in the OVX_P and OVX_B groups since the opposite relationship was expected. Additionally, it was surprising to find a positive correlation between femoral Ca concentrations and PTH concentrations. It appears that in the pumpkin and alendronate groups, the observed relationships are associated with the high accumulation of Ca in the kidneys. PTH stimulates Ca reabsorption in the kidneys and promotes its accumulation. The observed relationships undoubtedly have a multidirectional aspect. In ovariectomized rats with low estrogen levels, we expected adverse bone changes, but these rats were not Ca-deficient, and all diets had adequate amounts of Ca. As a result of changes in bones, we observed an increase in PTH, which influenced the kidneys by inhibiting Ca excretion, and Ca possibly was delivered to the bones via the action of other factors, such as ion shifts between tissues, or by biologically active substances of the drug or pumpkin components. Research suggests that the action of lycopene and carotenoids on bones is related to the activity of PTH (Burri et al. 2016). Moreover, obesity and increased bone marrow fat in the bones observed in ovariectomized rats might have an influence on the noticed changes in biochemical parameters. Other studies have shown a correlation between obesity and bone marrow fat and PTH activity (Rao et al. 2003; Fan et al. 2017). Weight gain in rats with low estrogen levels was expected, as was the increase in bone marrow fat, and this may possibly affect PTH levels in rats (Guasch et al. 2012), hence the lack of expected relationships between ovariectomy, bone Ca, and PTH. Unexpectedly, we did not observe significant changes (P > 0.05) in PINP and OC levels in ovariectomized groups. PINP and OC are nonspecific collagen proteins, mainly produced by osteoblasts, and their content in the blood can reflect the activity of osteoblasts (Guo et al. 2021). Although the number of osteoblasts increased in groups fed with diets containing pumpkin and alendronate alone and in combination, the relative increase in PINP level was only observed in OVX_P_B group, which may indicate an increased intensity of bone turnover due to the presence of two factors: bioactive ingredients of enriched pumpkin and alendronate. We also observed a link between the PINP level and changes in Ca in the body, as evidenced by the negative correlation between the PINP level and muscle Ca content. In the intervention groups after ovariectomy, Ca from the muscles was probably shifted to the bones and to the kidneys, as indicated by significant correlations (P < 0.05) between Ca content in these organs. Moreover, in ovariectomized rats, a significant decrease (P < 0.05) in Ca in the heart was observed, which might lead to problems with myocardial contractility. Other studies have confirmed that after ovariectomy, the sensitivity of Ca^2+^ myofilament is reduced, which leads to the release of Ca ions from the heart (Fares et al. 2013). An unexpected finding of this study is that the use of modified diets resulted in Ca accumulation in the kidneys. Unfortunately, no parameters of kidney functioning were analyzed, and histological analysis of the kidneys was not performed in this study. However, it can be assumed that Ca ions from other tissues were transported to the kidneys in the rats that received modified diets. In a study by Nijenhuis et al., a significant increase (P < 0.05) in the expression of TRPV5 (a protein responsible for the transport of Ca ions) was observed in bones following the administration of alendronate, while no such increase was observed in the kidneys and intestine (Nijenhuis et al. 2008). On the other hand, alendronate has been known to cause damage to the kidneys by forming Ca aggregates, which can lead to the formation of kidney stones or glomerulonephritis (Song and Maalouf 2000). Because the kidneys are responsible for the reabsorption of Ca, stones can restrict their filtration, resulting in hypercalciuria, and consequently, a decrease in Ca concentration in the blood ( Han et al. 2019). Enriched pumpkin contains ingredients that can affect kidney functioning. Inulin, which is one such ingredient, can expose the kidneys to a high amount of floating Ca due to its ability to increase Ca excretion (Adolphi et al. 2009). Large amounts of vitamins A and E found in pumpkins can lead to glomerular hyperfiltration and ultimately affect the filtration ability of the kidneys (Kedishvili 2016; Parente Filho et al. 2020; Chen et al. 2021).

For a detailed interpretation of the results, it is also worth paying attention to the results of histological analysis. In this study, the histopathological analysis of the femurs revealed interesting facts regarding bone cells, fat bone marrow degeneration, and woven bone. Osteoblasts are bone cells formed from mesenchymal precursors and eventually differentiate into osteocytes. Both osteoblasts and adipocytes are derived from the same stem cells, and thus a large amount of adipose tissue is an indicator of a large number of osteoblasts (Kos-Kudła et al. 2019). In this study, we observed a high number of both these cell types in ovariectomized rats; however, the increase in these cells was statistically significant (P < 0.05) only in the groups that received alendronate-supplemented diet, which suggests that stimulation of osteoblast differentiation intensifies the bone-building process (Ma et al. 2018). Rats with a high amount of adipose tissue also have a high percentage of adipose tissue marrow, as has been confirmed by previous studies on humans (Horowitz et al. 2017; van der Eerden and van Wijnen 2017) and animals (Iwaniec and Turner 2013; Fan et al. 2015). An interesting observation from these studies is the increased percentage of woven bone (immature bone) in the remodeling phase (Shapiro and Wu 2019). Woven bone is formed very quickly and appears porous. The proportion of woven bone is generally high during growth and puberty. On the other hand, in adults, this bone constitutes about 5–10%, while its higher share indicates structural overload or trauma, which is a temporary effect associated with the reconstruction of the lamellar (mature) bone (Hart et al. 2020). In this study, we observed that ovariectomy caused a significant increase (P < 0.05) in the percentage of woven bone, while the addition of enriched pumpkin and alendronate to the diet resulted in an opposite effect. Thus, it can be concluded that the reduction in estrogen levels led to the need for bone reconstruction in ovariectomized rats, as indicated by the increase in the percentage of woven bone in these animals. On the other hand, inulin and CaCO_3_ present in enriched pumpkin and alendronate accelerated bone reconstruction by increasing bone formation (discussed earlier), thus reducing the share of woven bone.

### Limitations

Due to several limitations of this study, some of the obtained results could not be highlighted here. Because rats’ urine was not collected in the study, we could not state whether its excretion increased with the accumulation of Ca in the kidneys. Other parameters related to bone metabolism, such as vitamins K and D, were not analyzed because only limited volume of serum was obtained from rats. The study also did not include a histological analysis of the kidneys, which could have been helpful in explaining the mechanism of Ca accumulation in this tissue. Furthermore, the study did not have a sham-operated control, and therefore the effect of sham surgery on rats was not analyzed; however, the results obtained in the ovariectomized group were compared with the nonoperated control group and the ovariectomized group fed with a standard diet. Unfortunately, we did not study the group with unenriched pumpkin, so we cannot determine what changes would occur in the rats’ organism if the pumpkin was not subjected to osmotic dehydration.

## Conclusion

CaCO3-enriched pumpkin can improve the concentration of Ca in the femur and bone recovery in ovariectomized rats, which is similar to the effect of alendronate. However, enriched pumpkin causes Ca accumulation in the kidneys, which is exacerbated when it is used in combination with alendronate. Further research is needed to elucidate the mechanism of calcium accumulation in the kidneys as a result of the consumption of calcium carbonate-enriched pumpkins.

## Data Availability

The data used to support the findings of this study can be made available by the corresponding author upon request.

## Abbreviations

PINP: Procollagen type I N propeptide
OC: Osteocalcin
PTH: Parathyroid hormone
ES: Estradiol
Ca: Calcium
CaCO_3_: Calcium carbonate
ELISA: Enzyme-linked immunosorbent assay
